# Latent profile analysis of resourcefulness among patients with type 2 diabetes and its relationship with self-management

**DOI:** 10.3389/fpubh.2025.1701150

**Published:** 2026-01-12

**Authors:** Shanshan Li, Nana Li, Hongxin Ni, Rui Qi, Dandan Zhang, Chunping Ni

**Affiliations:** 1 School of Nursing, Air Force Medical University, Xi'an, Shaanxi, China; 2 PLA No.96607 Hospital, Baoji, Shaanxi, China; 3 Qinghe Outpatient Department of Jingbei Medical Area of the General Hospital of the People's Liberation Army, Beijing, China; 4 Department of Cardiovascular Surgery, The Second Affiliated Hospital of Xi'an Jiaotong University, Xi'an, Shaanxi, China; 5 Department of Endocrinology, The First Affiliated Hospital of Air Force Military Medical University, Xi'an, Shaanxi, China

**Keywords:** influencing factors, latent profile analysis, resourcefulness, self-management, type 2 diabetes mellitus

## Abstract

**Objective:**

This study aimed to explore the potential categories of resourcefulness in patients with type 2 diabetes mellitus (T2DM) and their relationship with self-management.

**Methods:**

A total of 513 hospitalized T2DM patients in Xi’an were surveyed using a general information questionnaire, the Resourcefulness Scale, and the Diabetes Self-management Activities Scale for Type 2 Diabetes. Latent profile analysis (LPA) was conducted to identify resourcefulness subgroups, and their relationship with self-management was examined.

**Results:**

Patients with type 2 diabetes could be classified into three potential categories based on their resourcefulness status: a low resourcefulness level group (17.54%), a moderate resourcefulness level–social alienation group (66.66%), and a high resourcefulness level group (15.78%). Educational level, monthly per capita family income, and the presence or absence of complications were identified as factors influencing the potential categories of resourcefulness. Significant differences were observed in the total self-management scores among patients belonging to different potential categories of resourcefulness (*F* = 68.33, *p* < 0.001).

**Conclusion:**

Type 2 diabetes patients can be classified into three potential categories of resourcefulness, with those in the high resourcefulness level group demonstrating higher self-management ability. Improving the level of resourcefulness can contribute to enhancing patients’ self-management capabilities.

## Background

Type 2 diabetes mellitus (T2DM) is a chronic metabolic disorder characterized by high prevalence, increased disability and mortality rates, and significant healthcare costs (1), imposing a significant burden on individuals, families, and society as a whole (2). As a lifelong condition requiring continuous management, patients’ self-management ability is a critical determinant of effective disease control (3). Resourcefulness (4) refers to an individual’s ability to integrate internal and external resources to cope with life demands. This encompasses self-regulatory capacity to independently manage daily affairs (personal resourcefulness) and the proactive utilization of external support systems to obtain assistance when necessary (social resourcefulness). As a cognitive–behavioral skill, resourcefulness demonstrates distinct advantages in emotion regulation. When facing disease-related frustration and fear, it enables patients to maintain a relatively stable and positive psychological state. Furthermore, resourcefulness facilitates effective help-seeking behaviors, allowing patients to obtain adequate social support (5). These combined mechanisms significantly contribute to the effective implementation of self-management practices among patients. Previous studies have indicated (6–8) that low resourcefulness levels in chronic disease patients hinder the effective utilization of internal and external resources, thereby compromising self-management. However, existing research often evaluates resourcefulness solely based on scale scores, overlooking interindividual heterogeneity and resulting in non-targeted interventions. Latent profile analysis (LPA), a probabilistic model-based classification method, can effectively identify groups with similar characteristics (9), thereby improving intervention precision. This study employed LPA to explore latent classes of resourcefulness among T2DM patients and their association with self-management, aiming to provide evidence for tailored intervention strategies.

## Methods

### Study design and participants

A convenience sampling method was used to select 513 hospitalized patients from the endocrinology departments of one tertiary hospitals in Xi’an between December 2022 and June 2023 as study participants. The inclusion criteria for participants were as follows: (1) diagnosis of diabetes mellitus according to the WHO 1999 criteria (3), (2) diabetes duration ≥1 year, (3) age ≥18 years, (4) clear consciousness and the ability to complete the questionnaire independently, and (5) voluntary participation with signed informed consent. The exclusion criteria included the following: (1) presence of acute complications or impaired consciousness, (2) severe mental illness or use of psychiatric medications, and (3) participation in other clinical studies.

According to Kendall’s principle (10), the sample size should be 10 to 20 times the number of independent variables. Given the 16 independent variables in this study and an anticipated 20% rate of invalid responses, the target sample size was calculated to range from 108 to 216 participants. To achieve a good model fit effect, potential profile analysis usually requires a sample size of more than 500 cases (11). A total of 513 patients were ultimately included in the study.

## Measures

### Sociodemographic and clinical characteristics

Based on expert consultations, literature reviews, and previous studies, we designed a general demographic information questionnaire. It included items on gender, age, educational level, marital status, occupational status, monthly per capita family income (yuan), medical expense payment method, duration of illness (in years), presence of complications, and the current treatment method.

### Resourcefulness

The Chinese version of the Resourcefulness Scale (4) was used to evaluate individual resourcefulness, encompassing two dimensions: personal resourcefulness and social resourcefulness. It consists of 28 items, with 16 items measuring personal resourcefulness and 12 items measuring social resourcefulness, each scored on a 0–5 scale. A higher total score indicates greater resourcefulness. In the present study, the Cronbach’s alpha coefficient was found to be 0.89, indicating good internal consistency.

### Self-management

The Diabetes Self-management Activities Scale (12) was used to evaluate individual self-management. The scale consists of 11 items covering six dimensions: general diet, special diet, exercise, blood glucose monitoring, foot care, and medication adherence in diabetic patients. Each item is scored on a 0–7 scale, reflecting the number of days in the past week the behavior was performed. Higher total scores indicate better self-management. In the present study, the Cronbach’s alpha coefficient was found to be 0.74, indicating good internal consistency.

### Statistical analysis

Mplus 8.3 software was utilized for latent profile analysis (LPA), gradually increasing the number of categories and comparing fit indices between models to identify the best model based on both practical significance and statistical criteria. The primary fit indices included the Akaike information criterion (AIC), Bayesian information criterion (BIC), and the corrected Bayesian information criterion (aBIC), with smaller values indicating better model fit (13). Entropy ranges from 0 to 1, with values closer to 1 indicating more accurate classification (14). Model comparisons were conducted using the likelihood ratio test (LMR) and the bootstrap likelihood ratio test (BLRT), with *p*-values less than 0.01 considered statistically significant. A *p*-value of less than 0.05 indicated that the fit of the Kth model was superior to that of the K- 1th model (15).

Statistical analysis was conducted using SPSS 22.0 software. Measurement data were summarized using means and standard deviations or medians and interquartile ranges, while categorical data were presented as frequencies and percentages. For univariate analysis, a chi-squared (χ^2^) test or Fisher’s exact test was used for unordered categorical data. Multivariate logistic regression was performed to identify the factors influencing resourcefulness in patients with type 2 diabetes, with a significance level set at *α* = 0.05.

## Results

### Correlation analysis

The total resourcefulness score among type 2 diabetes mellitus (T2DM) patients was 78.62 ± 16.65, while the total self-management score was 42.15 ± 13.31. Correlation analysis revealed a positive association between resourcefulness and self-management in T2DM patients (*r* = 0.476, *p* < 0.001). Furthermore, all dimensions of resourcefulness were individually positively correlated with each dimension of diabetes self-management (*p* < 0.001 for all) (see Table 1).

**Table 1:** Correlation analysis between resourcefulness and self-management in patients with type 2 diabetes.

Item	Resourcefulness	Personal resourcefulness	Social resourcefulness	Self-management	Full diet	Special diet	Exercise	Glucose monitoring	Foot care	Pharmacy
Resourcefulness	1									
Personal resourcefulness	0.944**	1								
Social resourcefulness	0.919**	0.738**	1							
Self-management	0.476**	0.420**	0.473**	1						
Full diet	0.374**	0.349**	0.349**	0.599**	1					
Special diet	0.291**	0.251**	0.296**	0.544**	0.181**	1				
Exercise	0.310**	0.274**	0.308**	0.695**	0.204**	0.388**	1			
Glucose monitoring	0.217**	0.166**	0.247**	0.666**	0.324**	0.104*	0.317**	1		
Foot care	0.333**	0.293**	0.331**	0.677**	0.191**	0.262**	0.393**	0.353**	1	
Pharmacy	0.230**	0.227**	0.199**	0.469**	0.335**	0.157**	0.187**	0.227**	0.116**	1

**p < 0.01.

### Latent class analysis of resourcefulness

In models with 1 to 5 categories, the results indicated that as the number of categories increased, the values of the AIC, BIC, and aBIC decreased. In addition, the entropy value varied. Based on the fit indices and the practical significance of the potential categories, a three-category model was selected as the optimal solution (see Table 2).

**Table 2:** Model fit indices for latent profile analysis.

Profile	AIC	BIC	aBIC	Entropy	BLRT (*p*)	VLMR (*p*)	Sample size by profile based on most likely membership (%)
1C	42536.08	42773.54	42595.78				
2C	40404.36	40764.78	40494.98	0.86	<0.05	<0.05	49.12/50.87
3C	39281.98	39765.37	39403.52	0.94	<0.05	<0.05	15.78/17.54/66.66
4C	38971.79	39578.15	39124.25	0.87	0.49	0.49	10.33/12.08/34.43/43.27
5C	38818.06	39547.39	39001.43	0.88	0.30	0.30	6.23/11.89/15.01/22.02/44.83

AIC, Akaike information criterion; BIC, Bayesian information criterion; aBIC, adjusted BIC; LMR, Lo–Mendell–Rubin Adjusted Likelihood Ratio Test; BLRT, Bootrapped likelihood ratio test.

**Figure 1:**
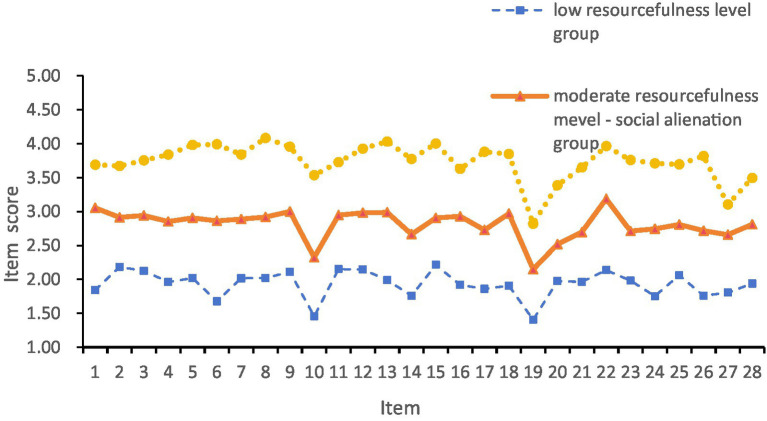
Potential categories of resourcefulness in patients with type 2 diabetes.

The results of the latent profile analysis (LPA) for the three categories are shown in Figure 1. The C1 group, comprising 17.54% of the T2DM patients, showed low scores across all dimensions and was therefore labeled the “Low Resourcefulness Level Group.” The C2 group, which scored between C1 and C3 across all items, showed notably lower scores on items 10 (“when I feel confused, I rely on others to help me”) and 19 (“if I do not have enough money to pay the bills, I will borrow money from someone”) in the social resourcefulness dimension. This group, accounting for 66.66% of the total participants, was therefore labeled the “Moderate Resourcefulness Level–Social Alienation Group.” The C3 group, comprising 15.78% of the T2DM patients, had the highest scores across all dimensions and was therefore labeled the “High Resourcefulness Level Group.”

### Univariate analysis of potential resourcefulness categories in T2DM patients

The results of the univariate analysis indicated that age, educational level, monthly per capita family income, and presence of complications differed significantly among the resourcefulness category groups (see Table 3).

**Table 3:** Univariate analysis of latent resourcefulness categories in T2DM patients.

Variable	Classification	Low resourcefulness level group	Moderate resourcefulness level–social alienation group	High resourcefulness level group	χ^2^	*p*
Age	23–44	5	34	20	13.43	0.015
	45–60	26	138	54		
	>60	59	170	7		
Educational level	Primary school	18	37	8	19.68	0.003
	Junior school	26	95	18		
	Senior school	27	100	15		
	College and above	19	110	40		
Income	<3,000元	70	56	0	244.15	<0.001
	300-5000元	12	150	6		
	>5000元	8	136	75		
Complications	Yes	24	178	66	51.35	<0.001
	No	66	164	15		

### Multifactorial analysis of potential resourcefulness categories in T2DM patients

A multivariate logistic regression analysis was performed using the variables that showed statistically significant differences in the univariate analysis as independent variables. Resourcefulness served as the dependent variable, and the “Low resourcefulness Level Group” was used as the reference group. The results indicated that being an undergraduate, having a monthly per capita family income exceeding 5,000 yuan, and the presence of complications were factors that differed significantly between the high and low resourcefulness level groups. As shown in Table 4, there was a statistically significant difference between the moderate resourcefulness level–social alienation group and the low resourcefulness level group in terms of having a monthly per capita family income greater than 5,000 yuan and the presence of complications.

**Table 4:** Multivariate logistic regression analysis of latent resourcefulness categories in T2DM patients.

Variable	Classification	High resourcefulness level group			Moderate resourcefulness level–social alienation group		
		*p*	OR	95%CI	*p*	OR	95%CI
Educational level	Junior school	0.042	0.29	0.088–0.957	0.621	1.304	0.456–3.73
	Senior school	0.941	1.048	0.307–3.577	0.317	1.723	0.594–5.002
	College and above	0.683	0.777	0.231–2.613	0.649	0.795	0.296–2.133
Income	3,000-5000元	0.178	2.455	0.665–9.056	0.359	1.425	0.669–3.033
	>5000元	<0.001	45.539	10.416–199.102	<0.001	14.907	5.195–42.773
Complications	yes	<0.001	0.093	0.043–0.202	<0.001	0.263	0.141–0.492

Educational attainment (dummy variables with “primary school and below” as the reference): Primary school and below: (0,0,0,0), junior high school: (0,1,0,0), senior high school/technical secondary school: (0,0,1,0), and undergraduate and above: (0,0,0,1). Monthly per capita household income (dummy variables with “< 3,000 RMB” as the reference): < 3,000 RMB: (0,0,0), 3,000–5,000 RMB: (0,1,0), and > 5,000 RMB: (0,0,1). Complications: Present = 0, absent = 1.

### Potential resourcefulness categories and self-management

The self-management scores of T2DM patients with different potential categories of resourcefulness were compared. The results showed that there was a statistically significant difference in the total self-management scores among these categories (*F* = 68.33, *p* < 0.001). The patients in the high resourcefulness group had the highest total self-management score (51.16 ± 12.19 points), whereas those in the low resourcefulness group had the lowest score (30.36 ± 13.03 points). The comparison of scores across each dimension among the three groups showed statistically significant differences (*p* < 0.001). The range of resourcefulness scores overlapped among three categories (see Table 5).

**Table 5:** Comparison of self-management scores among patients in different potential resourcefulness categories.

Item	Low resourcefulness level group	Moderate resourcefulness level–social alienation group	High resourcefulness level group	*F*	*p*
Self-management	30.46 ± 13.03	43.09 ± 11.42	51.16 ± 12.19	68.33	<0.001
Full diet	5.24 ± 3.67	7.55 ± 3.47	10.04 ± 2.85	42.02	<0.001
Special diet	5.02 ± 3.03	7.28 ± 3.00	7.91 ± 3.10	24.49	<0.001
Exercise	5.52 ± 3.53	8.44 ± 3.65	9.51 ± 3.86	29.68	<0.001
Glucose monitoring	6.81 ± 3.94	8.58 ± 4.01	9.39 ± 4.00	9.96	<0.001
Foot care	4.03 ± 3.26	6.09 ± 4.19	8.58 ± 4.19	26.94	<0.001
Pharmacy	3.83 ± 2.57	5.12 ± 2.27	5.71 ± 2.11	15.95	<0.001

## Discussion

In this study, the total resourcefulness score of T2DM patients was 78.62 ± 16.65. This finding is similar to the findings reported by Liang Qi et al. (16) in colorectal cancer patients, but it is higher than the scores noted by Li Xiumei et al. (17) in patients undergoing peritoneal dialysis. This discrepancy may be attributed to the unique challenges of T2DM as a chronic, lifelong condition, which is characterized by high prevalence, elevated disability and mortality rates, and substantial healthcare costs—all of which significantly impair patients’ quality of life. Lifelong treatment can also impose considerable psychological pressure and financial burden on patients and their families, necessitating enhanced cognitive and behavioral coping strategies to manage these pressures. The total self-management score of T2DM patients in this study was 42.15 ± 13.31, indicating a moderate to low level, consistent with previous research (18, 19). Importantly, our findings revealed a positive correlation between resourcefulness and self-management.

This study revealed heterogeneity in resourcefulness levels among type 2 diabetes mellitus (T2DM) patients, identifying three distinct latent classes: low resourcefulness group, moderate resourcefulness level–social alienation group, and high resourcefulness group. The low resourcefulness group (17.54%) exhibited low scores across all resourcefulness scale items, indicating poor disease cognition and limited ability to utilize social resources. Consistent with previous studies on other chronic conditions, this group demonstrated weaker self-management capabilities (8, 20). The moderate resourcefulness level–social alienation group (66.66%) scored between the low and high resourcefulness groups. The scale assessment revealed that patients scored acutely low on items measuring their ability to utilize external resources (Items 10: “when I feel confused, I rely on others to help me,” and 19: “if I do not have enough money to pay the bills, I will borrow money from someone”), indicating a pronounced difficulty in mobilizing external support systems within this cohort. These findings suggest that there is significant room for improvement through targeted interventions. The possible reasons can be analyzed as follows: First, the study included a relatively high proportion of older adults, who tend to have limited channels for acquiring knowledge. In addition, their inherent reluctance to burden others reduces their willingness to seek external help. Second, as a lifelong condition with a high incidence of complications, diabetes imposes a heavy burden that adversely affects patients’ psychological wellbeing. The high resourcefulness group (15.78%) achieved the highest scores across all dimensions, demonstrating strong internal and external resource utilization. This was associated with better self-management, higher life satisfaction, and improved disease control, aligning with evidence that social resourcefulness enhances diabetes outcomes. In addition, research has shown that fully leveraging external resources, such as social resourcefulness, contributes to better disease control in diabetic patients. Therefore, in clinical practice, attention should be paid to patients’ resourcefulness levels, and targeted interventions should be developed for those with low resourcefulness or poor social resource utilization to enhance their resourcefulness and self-management capabilities.

Regression analysis revealed that education level, monthly per capita household income, and the presence of complications were key factors influencing the latent classes of resourcefulness in patients with type 2 diabetes. The patients with lower education levels were more likely to belong to the low resourcefulness group. This may be because highly educated patients have better access to disease-related knowledge and coping strategies, enabling them to better understand the impact of the disease on their health and the importance of self-management (21). In this study, the patients with higher monthly per capita household income were more likely to belong to the moderate resourcefulness–social detachment group or the high resourcefulness group. As a lifelong condition, type 2 diabetes imposes a heavy financial burden on patients and their families due to prolonged treatment. Patients with higher incomes may invest more in their health and actively seek multiple sources of disease-related information. In addition, the patients with complications were more likely to belong to the moderate resourcefulness–social detachment group or the high resourcefulness group. A possible explanation is that the presence of complications provides patients with a more direct and clear awareness of their disease, prompting them to engage more actively in treatment once complications arise.

The findings of this study demonstrated significant differences in self-management levels among T2DM patients across distinct latent classes of resourcefulness. Notably, significant variations were observed across all dimensions of self-management among the three groups, indicating that resourcefulness broadly influences self-management capabilities in these patients. Patients in the low resourcefulness group had the lowest self-management scores, while those in the high resourcefulness group had the highest scores. Compared to the low resourcefulness group, the high resourcefulness group showed significantly higher scores across all dimensions of self-management, with the most pronounced improvements observed in diet (general diet and specialized), exercise, and foot care. This further confirms the positive correlation between resourcefulness and self-management in patients with type 2 diabetes. Therefore, implementing targeted interventions based on the demographic characteristics of patients is essential. For patients in the low resourcefulness group, repeated health education should be provided to continuously enhance their understanding of the disease. For patients in the moderate resourcefulness level–social alienation group, more avenues for accessing social resources and diversified social support information should be provided to strengthen their awareness of the importance of social resources. In addition, patients in the high resourcefulness group can be encouraged to share their experiential knowledge with peers, providing practical and effective support. Improving patients’ self-management abilities by enhancing their resourcefulness level is a feasible strategy. Furthermore, this study revealed overlapping resourcefulness score ranges across different latent classes, suggesting that patients with similar resourcefulness scores may belong to different latent categories. Healthcare providers should pay attention to individual differences and deliver personalized interventions tailored to the specific characteristics of each latent class to enhance the effectiveness and precision of clinical interventions.

In summary, the resourcefulness of T2DM patients can be categorized into three latent classes, and there is a positive correlation between resourcefulness and self-management. However, this study has some limitations. First, the representativeness of the sample is limited. All data were collected from a single medical center in Xi’an, and the results may be influenced by specific regional culture, medical practices, and population characteristics, which restricts the generalizability of the findings to patients in other regions or cultural contexts. Second, there is a notable selection bias. The study participants were all hospitalized patients, who generally have more severe conditions, higher complication rates, and greater challenges in self-management compared to T2DM patients managed in outpatient or community settings. Therefore, the sample does not represent the broader population of patients with milder conditions or more stable management. This may lead to biased estimates of the relationship between resourcefulness and self-management in the overall patient population, and caution is warranted when extrapolating the conclusions. Finally, the consideration of the influencing factors is incomplete. While analyzing the determinants of latent classes, the study primarily focused on demographic and clinical characteristics and did not systematically incorporate important variables such as socioeconomic status, psychological state, social support, and healthcare accessibility. This may limit the in-depth understanding of the underlying causes of resourcefulness classification and its association with self-management. Therefore, future research should expand geographical coverage and increase the sample size while incorporating additional potential influencing factors for a more comprehensive investigation. In addition, the cross-sectional design cannot establish causal relationships between resourcefulness and self-management. Therefore, it is recommended that subsequent studies employ longitudinal approaches to further examine and verify the relationship between resourcefulness and self-management over time.

## Data Availability

The original contributions presented in the study are included in the article/supplementary material, further inquiries can be directed to the corresponding author.
